# Sensitive Determination of Terazosin in Pharmaceutical Formulations and Biological Samples by Ionic-Liquid Microextraction Prior to Spectrofluorimetry

**DOI:** 10.1155/2012/546282

**Published:** 2012-02-08

**Authors:** Mohsen Zeeb, Mahdi Sadeghi

**Affiliations:** ^1^Agricultural, Medical and Industrial Research School, Nuclear Science and Technology Research Institute, P.O. Box 31485/498, Karaj, Iran; ^2^Department of Analytical Chemistry, Faculty of Chemistry, University College of Science, University of Tehran, P.O. Box 14155-6455,Tehran, Iran

## Abstract

An efficient and environmentally friendly sample preparation method based on the application of hydrophobic 1-Hexylpyridinium hexafluorophosphate [Hpy][PF_6_] ionic liquid (IL) as a microextraction solvent was proposed to preconcentrate terazosin. The performance of the microextraction method was improved by introducing a common ion of pyridinium IL into the sample solution. Due to the presence of the common ion, the solubility of IL significantly decreased. As a result, the phase separation successfully occurred even at high ionic strength, and the volume of the settled IL-phase was not influenced by variations in the ionic strength (up to 30% w/v). After preconcentration step, the enriched phase was introduced to the spectrofluorimeter for the determination of terazosin. The obtained results revealed that this system did not suffer from the limitations of that in conventional ionic-liquid microextraction. Under optimum experimental conditions, the proposed method provided a limit of detection (LOD) of 0.027 *μ*g L^−1^ and a relative standard deviation (R.S.D.) of 2.4%. The present method was successfully applied to terazosin determination in actual pharmaceutical formulations and biological samples. Considering the large variety of ionic liquids, the proposed microextraction method earns many merits, and will present a wide application in the future.

## 1. Introduction

Terazosin is a selective potent *α*_1_ adreno-receptor antagonist. It is an effective drug for hypertension and benign prostatic hyperplasia [1, 2]. The needs of monitoring trace levels of terazosin in treated patients with initiate or chronic treatment routine in chronic regimen, especially in renal impairment cases, are necessary. To prevent excess employed dosage, it is critical to develop the sensitive and accurate techniques for its quantitative analysis. Several analytical methods have been developed for the measurement of terazosin in bulk, pharmaceuticals, or biological fluids [3–8].

Sample preparation is a critical step to isolate the analytes of interest from the sample matrix as well as to increase the concentration of analytes prior to their determination, especially when their levels are lower than the detection limit of analytical techniques. Liquid-liquid extraction (LLE) and solid-phase extraction (SPE) have been widely used as basic, simple, and adaptable methods for sample preconcentration. However, some drawbacks such as the use of large amount of hazardous organic solvents, and the employment of multistep procedures associated with the high risk of analyte losses make LLE and SPE tedious, expensive, and environmentally unfriendly. In recent years, reduction or complete removal of hazardous organic solvents in sample pretreatment techniques has received great attention. For this goal, many microextraction methods such as dispersive liquid-liquid microextraction (DLLME) [9–12], single-drop microextraction (SDME) [13–15], stir bar sorptive extraction [16], and so forth, have been developed to reduce the amount of toxic solvents. In some of these procedures, however, the use of toxic materials like benzene, toluene, or chloroform as the microextraction solvent is a common practice.

Ionic liquids (ILs) are ionic media resulting from combinations of organic cations and different anions. ILs have a variety of chemical and physical properties such as low vapor pressure, excellent thermal stabilities, adjustable miscibility, moderate solubility of organic compounds and metal ions, and so forth, which make them highly attractive in microextraction methods [17]. ILs have been widely applied in many sample pretreatment procedures as green extraction solvent including ionic liquid-based liquid-liquid microextraction (IL-LLME) [18, 19], cold induced aggregation microextraction (CIAME) [20], ionic liquid-based single-drop microextraction (IL-SDME) [21, 22], ionic liquid-based dispersive liquid-liquid microextraction (IL-DLLME) [23, 24], in situ solvent formation microextraction (ISFME) [25], temperature-controlled ionic liquid dispersive liquid phase microextraction (TILDLME) [26], and so forth.

The use of separation and preconcentration steps based on IL-based microextraction methods offers several advantages such as high recovery, no need of hazardous extraction solvent, simplicity of operation, and compatibility with many analytical techniques. Despite the many advantages of using ionic liquid-based microextraction techniques, under high salt condition, the fine droplets of the extraction phase can not be settled due to increase in sample solution density. The solubility of ionic liquids increases as the salt amount of sample increases. As a result, the volume of the settled phase depends significantly on the ionic strength of the sample solution. On the other hand, accuracy and performance of the extraction method are significantly influenced by variations in the ionic strength. In this work, in order to solve these problems and obtain higher accuracy, a novel, simple, and powerful ionic-liquid microextraction method which put very few dangers to environment was developed. In the proposed approach, a hydrophobic pyridinium IL was added to the aqueous sample solution containing one of the IL's ions as a common ion to act as the extraction phase. Hydrophobic 1-hexylpyridinium hexafluorophosphate [Hpy][PF_6_] ionic liquid and NaPF_6_ were used as the extraction phase and common ion source, respectively. Due to the presence of the common ion in the sample, the solubility of IL decreases, even at high ionic strength. Therefore, the volume of the enriched phase is independent from the ionic strength. In this work, [Hpy][PF_6_] was employed as the extractant solvent due to some physicochemical properties such as high hydrophobicity, water immiscibility, and suitable density. The present methodology does not need heating, antisticking agent, long equilibration time, and cooling before or after centrifugation prior to analysis. To the best of our knowledge, up until now, no method based on the microextraction with ILs and spectrofluorimetric detection has been proposed for preconcentration, extraction, and determination of drugs in their trace levels. Spectrofluorimetric method was applied due to ease, proper selectivity, sensitivity, wide dynamic range, and low cost of operation. The goal of this paper is to study the applicability of the proposed microextraction method followed by spectrofluorimetry to determine terazosin as a model compound in pharmaceutical and biological samples. Furthermore, the obtained results reveal that the proposed methodology is simple and practical for the routine determination of terazosin in aqueous media.

## 2. Experimental

### 2.1. Instrumentation

Fluorescence measurements were performed using a Perkin-Elmer LS 50 spectrofluorimeter equipped with xenon discharge lamp, and quartz micro-cell with a volume of 100 *μ*L. A centrifuge from Hettich (Tuttlingen, Germany) was utilized to accelerate the phase separation process. An adjustable sampler (10–100 *μ*L) was prepared from Eppendorf (Hamburg, Germany). The pH-meter model 692 (Herisau, Switzerland) supplied with a glass-combined electrode and universal pH indicator (pH 0–14) from Merck (Darmstadt, Germany) were used for the pH measurements.

### 2.2. Reagents and Materials

All chemicals used were of analytical reagent grade and all solutions were prepared with ultrapure water. 1-Hexylpyridinium hexafluorophosphate [Hpy][PF_6_] (97%) (Acros organics, Belgium) was applied as an extractant solvent. Acetone, acetonitrile, methanol, ethanol and sodium hexafluorophosphate (NaPF_6_) were purchased from Merck (Darmstadt, Germany). Stock solution of terazosin at concentration of 1000 mg L^−1^ was prepared by dissolving the required amount of pure drug in ultrapure water, and working standard solutions were obtained by serial dilutions of this stock solution. [HPy][PF_6_] IL is not liquid at room temperature (melting point: 45°C). Therefore, this IL was dissolved in acetonitrile to obtain a working solution of 75 mg mL^−1^. A solution of 250 mg mL^−1^ NaPF_6_ was obtained by dissolving appropriate amount of this salt in ultrapure water. 0.5 mol L^−1^ of sodium hydroxide and concentrated hydrochloric acid were used for adjusting the pH value of sample solutions. Terazosin tablets (labeled as containing 2 and 5 mg terazosin per tablet) were obtained from Daroupakhsh (Iran, Tehtan).

### 2.3. General Analytical Procedure

Aliquots of 10.0 mL sample or standard solution (pH 10) containing terazosin in the range of 0.1 to 115 *μ*g L^−1^ was placed into a glass test tube with conic bottom, 1.4 mL of NaPF_6_ (250 mg mL^−1^) was added. Then, 150 mg of [HPy][PF_6_] (dissolved in acetonitrile) as the extraction solvent was added to the sample solution. After shaking, a cloudy condition was immediately formed at room temperature. Due to the formation a large contact area between extraction solvent and analyte, the uncharged form of terazosin was rapidly trapped into IL-phase. In order to accelerate phase separation, the resultant solution was centrifuged for 5 min at 4000 rpm. The upper aqueous solution was then removed by a pipette. Finally, in order to reduce the viscosity of IL-phase and facilitate sample handling prior to analysis, the settled phase (about 16 *μ*L) in the test tube was diluted to 250 *μ*L by adding appropriate amount of ethanol. 100 *μ*L of the diluted settled phase was removed by a sampler and transferred to the microcell of the fluorimeter. The fluorescence intensity was measured at 376 nm with the excitation wavelength set at 330 nm. A reagent blank was prepared using a similar procedure but without adding terazosin.

### 2.4. Analysis of Tablets

Five terazosin tablets, labeled as containing 2 mg terazosin each, were weighed and the average mass per tablet was determined. An amount of the powder equivalent to 2 mg of terazosin was accurately weighed and dissolved in 50 mL ultrapure water. The solution was sonicated for 5 min and filtered into a 100 mL volumetric flask. Further dilutions were made up with ultrapure water to achieve a final concentration of 20 *μ*g mL^−1^. In addition, appropriate dilution was performed for terazosin analysis.

### 2.5. Analysis of Spiked Human Plasma

Human plasma samples (1.0 mL) were spiked with terazosin solutions, deproteinized by adding 4 mL acetonitrile, and centrifuged at 4000 rpm for 15 min. Then, 2.0 mL of the clear supernatant was diluted to 50 mL and subjected to the proposed method.

### 2.6. Analysis of Spiked Human Urine

Fresh matutinal human urine (10 mL) was transferred into graduated centrifuge tubes. These solutions were centrifuged for 5 min at 4000 rpm. Then, aliquots of 2 mL from clear supernatant were put in new centrifuge tubes and spiked with different amounts of terazosin (2 to 100 *μ*g L^−1^); the general analytical procedure was followed.

## 3. Results and Discussion

In this work, a practical and simple ionic-liquid microextraction method was combined with spectrofluorimetry for preconcentration and determination of terazosin. Hydrophobic [Hpy][PF_6_] and NaPF_6_ were applied as the extraction phase and common ion source, respectively. In the proposed microextraction method, the volume of the enriched phase was independent form the ionic strength of the sample solution (up to 30% w/v) which as an advantage significantly improved the performance of the extraction method. The influence of different factors affecting extraction conditions were studied and optimized to obtain a compromise between sensitivity, simplicity, and reproducibility.

### 3.1. Spectral Characteristics of Terazosin and [HPy][PF_6_]

**Figure 1:**
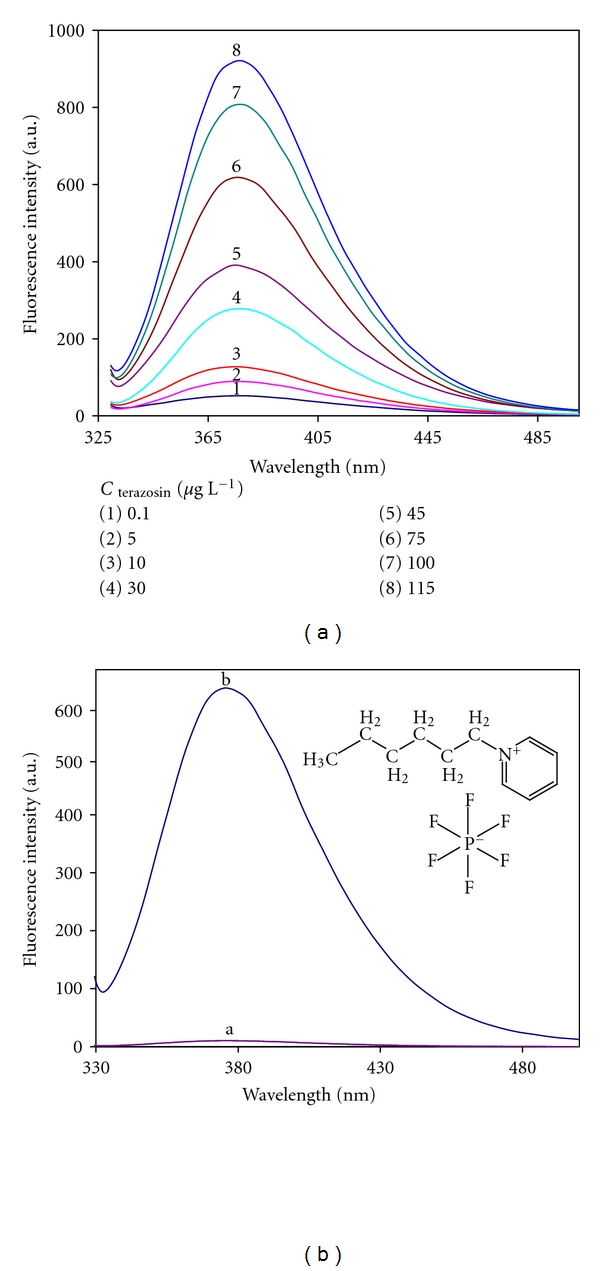
(a) Emission spectra obtained for 8 standard solutions of terazosin with different concentrations. (b) Emission spectrum of terazosin (75 *μ*g L^−1^) treated the same as previously described in the general analytical procedure (a), and emission spectrum of reagent blank in [Hpy][PF_6_] ionic liquid (b). Inset: chemical structure of [Hpy][PF_6_] ionic liquid. Experimental conditions were as indicated in the Table 1.

The intense fluorescence of terazosin is due to its cyclic conjugated structure which benefits of having *π*-electron system. The emission spectra of terazosin were recorded as described in the general analytical procedure (see Figure 1(a)). As it can be ascertained, the emission peak of terazosin is at 376 nm, while its excitation peak is at 330 nm. In order to obtain accurate fluorescence intensity, it is important to study the effect of the extraction phase on fluorescence spectrum of the analyte of interest. For this goal, fluorescence spectrum of the reagent blank was evaluated. As it can be seen in Figure 1(b), the emission of [HPy][PF_6_] has no notable effect on the determination of terazosin. Thus, the aforementioned wavelengths were selected for quantitative analysis of terazosin.

### 3.2. Influence of IL Amount

**Figure 2:**
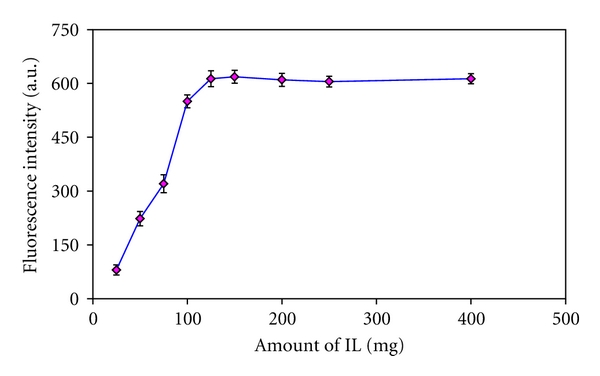
Influence of amount of [Hpy][PF_6_] on the fluorescence intensities. Experimental conditions: terazosin concentration 75 *μ*g L^−1^; pH 10; NaPF_6_ 350 mg.

The amount of [HPy][PF_6_] is a critical parameter which can affect the analyte recoveries. Thus, the microextraction procedure was carefully evaluated in order to achieve a compromise between the amount of IL and the analytical responses. The Effect of [HPy][PF_6_] amount on the analytical responses was studied within the range of 25–400 mg.  Figure 2 shows the variation of the fluorescence signal versus the amount of [HPy][PF_6_]. As it can be ascertained, the signal intensity increases as the amount of [HPy][PF_6_] increases and remains nearly constant from 125 mg. No significant variations were obtained on the microextraction efficiency for higher amounts of IL. Thus, in order to achieve sensitive analytical response, 150 mg of IL was selected as an optimum value.

### 3.3. Influence of Common Ion

**Figure 3:**
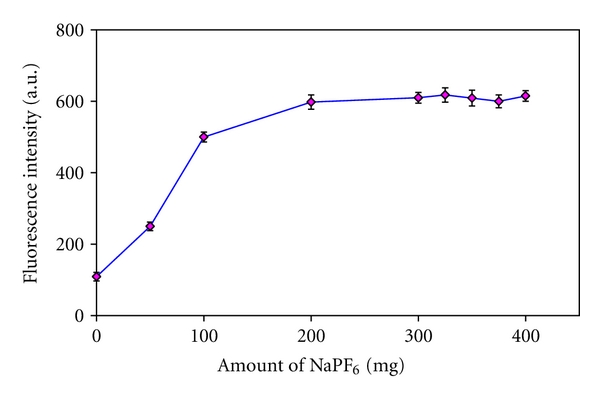
Influence of NaPF_6_ on the analytical signals obtained for terazosin. Experimental conditions: terazosin concentration 75 *μ*g L^−1^; pH 10; [Hpy][PF_6_] 150 mg.

The effect of NaPF_6_ amount as a common ion source on the fluorescence signal was studied within the range of 0–400 mg (see Figure 3). Due to the common ion influence, an increase in the amount of NaPF_6_ causes a notable decrease in the solubility of [HPy][PF_6_]. As a result, the extraction performance and the analytical intensity increase. As it can be seen in Figure 3, the fluorescence intensity increases with increasing the amount of NaPF_6_ up to 325 mg and then remains nearly stable. However, 350 mg of NaPF_6_ was chosen through the rest of the work.

### 3.4. Influence of Diluting Agent

Due to the high viscosity of the IL, it must be conditioned with a diluting agent prior to its introduction to the spectrofluorimeter. Several diluting agents including methanol, ethanol, acetone, and acetonitrile were evaluated in order to choice a diluting agent which can dissolve the IL-phase completely and provide the best sensitivity. Type of the organic solvent had no significant effect on the analytical response. Finally, due to less toxicity of ethanol, it was applied as diluting agent in all experiments.

### 3.5. Influence of pH

**Figure 4:**
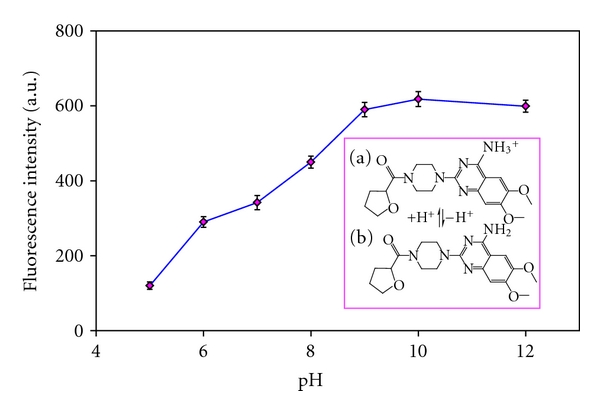
Influence of pH on the fluorescence intensities. Experimental conditions: terazosin concentration 75 *μ*g L^−1^; [Hpy][PF_6_] 150 mg; NaPF_6_ 350 mg. Inset: chemical structure of terazosin. Acid base equilibrium involved at working pH. (a) Protonated form of this terazosin; (b) dissociated form of terazosin.

The pH of the sample is an important factor which affects the chemical structure of terazosin. The effect of pH on the extraction of terazosin from aqueous samples was studied over the range of 5–12 using HCl and NaOH. For ionizable organic molecules, maximum extraction efficiency is obtained at pH values where the uncharged form of the analyte overcomes, and therefore, the analyte of interest is favored to be partitioned into the hydrophobic IL-phase. The results illustrated in Figure 4 reveal that analytical signals, obtained for terazosin, depend on pH. The best conditions of terazosin microextraction were achieved at alkaline pH values due to dissociation of the aromatic amine group, which acts as a weak base (see Figure 4) (pK_b_ = 6.9). Based on the results obtained, pH 10 was selected as the optimum value.

### 3.6. Influence of Equilibration Temperature and Extraction Time

In order to obtain complete extraction of terazosin and easy phase separation, the effect of equilibration temperature was studied from 5 to 40°C. Based on the results obtained in this study, the temperature had no significant and benefic effect on the extraction efficiency. Therefore, room temperature as an equilibration temperature was used through the rest of the work. Microextraction time is one of the important factors affecting the extraction efficiency, especially in microextraction methods such as SPME and LPME. The dependence of extraction efficiency upon extraction time was studied from 5 sec to 30 min. The results obtained in this experiment revealed that signal variations versus extraction time were not significant. It was demonstrated that after formation of the cloudy condition, the surface area between extraction phase and aqueous media was infinitely large. Thus, the transfer of the target analyte from aqueous media to IL-phase was very fast. In order to keep extraction time as short as possible, the cloudy solution was centrifuged immediately after the preparation.

### 3.7. Influence of Centrifugation Condition

The effect of centrifugation rate on the fluorescence intensity was evaluated from 1000 to 6000 rpm. The obtained results showed that over 3500 rpm the IL-phase was completely transferred to the bottom of the test tube, and the analytical response remained constant. Thus, 4000 rpm was selected for all experiments. At the optimum rate, the effect of centrifugation time upon analytical responses was investigated within the range of 1–15 min. Over 4 min, the fluorescence intensity remained stable indicating entire transfer of IL-phase to the bottom of the centrifuge tube. Therefore, a centrifugation time of 5 min was chosen as the optimum value.

### 3.8. Influence of Salt Concentration

Influence of salt concentration on the extraction performance was studied by adding various amounts of NaNO_3_ from 0 to 40% (w/v) while other experimental conditions were kept constant. In the proposed microextraction method, due to the presence of PF_6_^−^ as a common ion in the sample solution, the volume of the enriched phase was independent from the ionic strength, and phase separation occurred up to 30% w/v of NaNO_3_.  Table 1 summarizes both instrumental and experimental conditions selected.

### 3.9. Influence of Interfering Substances

The effect of different chemical species on the determination of target analyte was studied using solutions containing 75 *μ*g L^−1^of terazosin, and adding 100 mg L^−1^ of the possible interfering species. The tolerance limit of each interfering substance was taken into account as the largest amount yields an error in the determination of the target analyte which does not exceed 5%. No interference was observed from commonly interfering species such as Na^+^, NH_4_^+^, Ca^2+^, Zn^2+^, Mg^2+^, Cl^−^, PO_4_^3−^, SO_4_^2−^, starch, glucose, lactose, fructose, sucrose, ascorbic acid, citric acid, dye species (as yellow quinoline), urea, and saccharin. The obtained results revealed the excellent selectivity of the proposed methodology in detecting the studied drug in dosage form and biological samples.

## 4. Application

### 4.1. Analytical Performance

Under the optimum conditions, calibration graph was achieved by analyzing 10.0 mL of standard solutions containing known amounts of terazosin. The settled ionic phase was diluted to 250 *μ*L with ethanol and the fluorescence intensity was recorded. Therefore, a preconcentration factor of 40 was achieved. The enhancement factor calculated as the slope ratio of calibration graph after and before extraction was about 33. The linear concentration range was from 0.1 to 115 *μ*g L^−1^ of terazosin with linear regression equation as

(1) I  =  51.33  +  7.5608  C,

where *I* is the fluorescence intensity at 376 ± 3 nm and *C* is the terazosin concentration in *μ*g L^−1^. The relative standard deviation (R.S.D.) obtained for the determination of 75 *μ*g L^−1^ of terazosin was 2.4% (*n* = 5). The limit of detection (LOD), calculated as three times the standard deviation of the measurement of blanks divided by the slope of the calibration curve, was found to be 0.027 *μ*g L^−1^.

### 4.2. Analysis of Terazosin in Pharmaceutical Formulations

In order to show the validity of the proposed method, it was applied for terazosin determination in commercial tablets. Three replicate determinations were performed, and satisfactory results were achieved.  Table 2 shows the results obtained by applying the present method and those obtained by a reported voltammetric method [8]. Until date no official method has been reported for terazosin determination. The results show the applicability of the proposed methodology and its accuracy for quantitative analysis of terazosin in this type of samples.

### 4.3. Analysis of Terazosin in Spiked Human Plasma and Urine

The accuracy of the present method was evaluated by determination of terazosin in spiked human urine and spiked human plasma. The recovery of the studied drug was investigated at four concentration levels. The obtained results are shown in Table 3. As can be seen, calculated amounts of recoveries varied between 91.5–105.8% and 86.5–96.0% for human urine and human plasma, respectively, indicating both accuracy and precision.

### 4.4. Comparison with Other Reported Methods

Determination of terazosin by the proposed methodology was compared with other reported techniques. The results are shown in Table 4. As it can be seen, compared to the previously reported methods, the present method has a relatively low LOD, wide dynamic range, and excellent reproducibility. The method developed in this work is proposed as a suitable alternative to expensive instrumental methods for trace determination of terazosin in pharmaceutical and biological samples. These results reveal that the proposed approach is a very sensitive, low-cost, rapid, environmentally friendly and accurate technique that can be used for terazosin determination in routine analytical laboratories.

## 5. Conclusion

A novel and efficient mode of ionic-liquid microextraction was combined with spectrofluorimetric detection to preconcentrate and determine trace levels of terazosin. Hydrophobic [Hpy][PF_6_] ionic liquid was applied as a green extraction solvent and an alternative to hazardous organic solvents. Due to the presence of one of the IL's ions as a common ion in the sample solution, the performance of the microextraction method was not influenced by variations in the ionic strength of the aqueous solution (up to 30%). The method developed in this work was demonstrated to be simple, rapid, practical, inexpensive, and environmentally friendly for preconcentration and separation of the trace analytes. In addition, the present method revealed to be a practical tool for routine quality control of drugs in pharmaceutical and biological samples with low operation cost and simplicity of instrumentation.

## Figures and Tables

**Table 1:** The experimental conditions for terazosin determination.

Microextraction parameters	Studied range	Selected condition
Amount of NaPF_6_ (mg)	0–400	350
Amount of [Hpy][BF_4_] (mg)	25–400	150
pH	5–12	10
Equilibration temperature (°C)	5–40	Room temperature
Centrifugation time (min)	1–15	5
Ionic strength (% w/v)	0–40	NE^a^

Instrumental parameters		
Excitation wavelength (nm)	200–350	330
Emission wavelength (nm)	350–700	376
Excitation and emission slit widths (nm)	5–20	10

^a^No effect.

**Table 2:** Determination of terazosin in tablets by the proposed methodology and by a reported voltammetric method [8].

Claimed (mg/tablet)	Proposed methodology (mg)^a^	Reported method (mg)^a^	Error (%)^b^	Error (%)^c^
2	1.95 (±0.08)	2.07 (±0.10)	−2.5	−5.8
5	5.1 (±0.21)	5.27 (±0.37)	+2.0	−3.2

^a^Values in parenthesis show the standard deviation based on three replicates.

^b^Error against the declared value.

^c^Error against the reported method.

**Table 3:** Determination of terazosin in spiked urine and spiked plasma by present work.

Drug		Spiked urine			Spiked plasma	
	Amount added (*μ*g L^−1^)	Amount found (*μ*g L^−1^) ± S.D.^a^	Recovery (%)	Amount added (*μ*g L^−1^)	Amount found (*μ*g L^−1^) ± S.D.^a^	Recovery (%)
Terazosin	2	1.83 ± 0.09	91.5	2	1.73 ± 0.11	86.5
	5	4.81 ± 0.28	96.2	5	4.82 ± 0.33	96.4
	10	9.40 ± 0.30	94.0	10	9.60 ± 0.51	96.0
	100	105.81 ± 4.11	105.8	100	93.10 ± 4.88	93.1

^a^Average of three independent measurements.

**Table 4:** Comparison of the proposed approach with other reported methods for determination of terazosin.

Method	Sample	LOD (*μ*g L^−1^)	R.S.D. (%)	LR (*μ*g L^−1^)	References
Spectrofluorimetry	Serum, urine	210	2.5	Up to 3486.9	[4]
X-ray fluorescence spectrometry	Drug	732	—	732–843000	[5]
HPLC with fluorescence detection	Plasma	0.25	<7	Up to 100	[6]
HPLC with electrospray ionization mass spectrometry detection	Plasma	0.0625	—	—	[7]
Square-wave adsorptive cathodic stripping voltammetry	Plasma, drug	0.0058	<1.1	0.39–11.62	[8]
ionic-liquid microextraction spectrofluorimetry	Urine, plasma, drug	0.027	2.4	0.1–115	This work

LOD: limit of detection, LR: linear range, R.S.D.: relative standard deviation.
